# Environmental Lead Exposure among Preschool Children in Shanghai, China: Blood Lead Levels and Risk Factors

**DOI:** 10.1371/journal.pone.0113297

**Published:** 2014-12-01

**Authors:** Jia Cao, Minming Li, Yu Wang, Guangjun Yu, Chonghuai Yan

**Affiliations:** 1 Ministry of Education-Shanghai Key Laboratory of Children's Environmental Health, Xinhua Hospital Affiliated to Shanghai Jiao Tong University School of Medicine, Shanghai, China; 2 Children's Hospital of Shanghai, Shanghai, China; Nottingham University, United Kingdom

## Abstract

**Objective:**

To determine blood lead levels and to identify related risk factors among children in Shanghai; to explore the lead change trend of children after industrial transformation and to provide data for policy development to control environmental lead pollution in Shanghai.

**Methods:**

A stratified-clustered-random sampling method was used. A tungsten atomizer absorption spectrophotometer was employed to determine blood lead levels.

**Results:**

The arithmetic mean, geometric mean and median of blood lead levels of 0- to 6-year-old children from Shanghai were 22.49 µg/L, 19.65 µg/L and 19.5 µg/L, including 0.26% (6/2291) with concentrations ≥100 µg/L and 2.7% (61/2291) with concentrations ≥50 µg/L. Boys' levels (23.57 µg/L) were greater than those of girls (21.2 µg/L). The blood lead levels increased with age. This survey showed that the Chongming district was the highest and Yangpu district was the lowest, this result is completely opposite with the earlier survey in Shanghai. Risk factors for lead contamination included housing environment, parents' education levels, social status, hobbies, and children's nutritional status.

**Conclusions:**

The blood lead levels of children in Shanghai were lower than the earlier data of Shanghai and those of published studies in China, but higher than the blood lead levels of developed countries. The blood lead levels of urban districts are higher than the central districts with the industrial transformation. Society and the government should take an active interest in childhood lead poisoning of urban areas.

## Introduction

Lead is a widespread non-degradable pollutant that accumulates in the environment. It can contaminate the food chain, soil, water and air, leading to human disease [1]. Lead has a negative impact on nervous, hematopoietic, digestive, urinary, reproductive, cardiovascular, endocrine, immune, and skeletal system function. However, the main effects are seen in the nervous and hematopoietic systems. Lead is absorbed and spread through the blood, resulting in long-term accumulation in the liver, kidney, spleen, lung, and brain. Absorbed lead is tightly adherent to contaminated cells, negatively impacting intelligence, brain development, hemoglobin formation, and childhood growth [2]. Children lead uptake, absorption rates and accumulation are generally higher than those of adults.

Work in Europe, the United States, and other developed countries has demonstrated the harm of low lead levels to children's physical health, mental health, growth, and development [3]. Lanphear BP [4] reported that children's Intelligence Quotient (IQ) was negatively impacted by blood lead levels (BLL) ≥100 and <100 µg/L. The greatest impact on IQ was seen with blood lead levels in the 50∼100 µg/L range. America and other developed countries monitor childhood lead levels, leading to a sharp decrease in lead poisoning.

The average blood lead levels of Chinese urban children from 0 to 6 years of age decreased from 70–100 µg/L to 25–60 µg/L over the period from the end of the 1990s until 2009 [5], [6]. The prevalence of children with higher blood lead levels (≥100 µg/L) decreased from 30–50% to 1.5–15% [7], [8]. According to the earlier surveys of our team in Shanghai, the blood lead level decreased from 83 µg/L to 38.08 µg/L from 1997 to 2008.Our early survey included five districts, they were Jingan, Jiading, Chongming, Yangpu and Xuhui. The blood lead levels declined 7 µg/L, 3 µg/L, 2 µg/L, 2 µg/L and 4 µg/L from 1997 to 1999, respectively [9], [10]. The decline in blood lead levels appears to be associated with national efforts to decrease lead pollution, including the phase out of lead gasoline, a transition from coal fuel to diesel, natural gas, and other clean energy alternatives, and close or merge heavily polluted enterprises. Many lead-polluting industries have migrated from large cities to middle-size and small cities or to rural areas, or from eastern China to western China. There are seventeen years passed away since the introduction of lead free gasoline in Shanghai, few studies have reported the changes of lead levels and the associated factors in children.

## Subjects and Methods

### Subjects

This study was conducted in Shanghai City, China, from June 2013 to July 2014. A stratified-clustered-random sampling method was used in the survey. The Jingan, Jiading, Chongming, Yangpu, Xuhui, and Pudong districts were selected as the areas for investigation. Fifty 0- to 3-year-old children and seventy 3- to 6-year-old children were random selected for every age range. Written informed consent was acquired from each child's parents. The study was approved by the Medical Ethics Committee of Xinhua Hospital affiliated to Shanghai Jiao Tong University School of Medicine. 1991 America Center for Disease Control criteria for blood poisoning (BLL ≥100 µg/L) and low lead exposure (BLL ≥50–100 µg/L) were used.

### Questionnaire

A 30-min interview questionnaire was administered to each parent of children. The questionnaire characterized family characteristics, living environment, living habits, health and dietary habits of the tested children.

### Methods

Sample collection and storage were performed in a clean room with no obvious smoke and dust, at a location distant from any source of pollution. Nurses and medical staff were required to cut their nails short and wash their hands with a 2% EDTA (Na2) tampon before blood sampling. A 75% alcohol cotton ball was then used to disinfect the skin. Children's hands were cleaned with soap and water, dried, and 3 ml venous blood was then collected.

Samples were immediately placed in refrigeration until testing. A tungsten atomizer absorption spectrophotometer (PinAAcle900Z, USA) was used to determine blood lead levels. This method directly measures blood lead levels and is simple and rapid. No pretreatment is required, minimizing the chance of contamination.

### Quality control

All sampling equipment and containers were tested and found to be lead-free. Experienced staff nurses performed blood sampling. The collection courses were accurate. In the process of detection of lead, the quality control according with the sampling test is needed in order to ensure the quality of sample. Blood lead levels greater than 100 µg/L were retested and the average reported.

### Statistical analysis

Descriptive statistics were used to describe the study population. The means and median of lead levels were calculated. The association between lead levels and demographic factors were compared using ANOVA (for normally distributed data) or the Kruskal-Wallis test (for non-normally distributed data). A logistic regression was used to analyze the correlation between lead levels and risk factors. Statistical analyses were carried out using SPSS 18.0 for Windows (SPSS Inc., Chicago, IL, USA). P<0.05 (two-tailed test) was considered statistically significant.

## Results

The main characteristics of the children evaluated were shown in Table 1. The arithmetic mean, geometric mean and median of blood lead levels of 0- to 6-year-old children from Shanghai were 22.49 µg/L, 19.65 µg/L and 19.5 µg/L, including 0.26% (6/2291) with concentrations ≥100 µg/L and 2.7% (61/2291) with concentrations ≥50 µg/L.

**Table 1:** General characteristics.

Characteristics	
Boy/Girl	1186/1105
Age (months)	
0–11	9.6% (221)
12–35	31% (710)
36–72	59.4%(1360)
Jingan district	17.2% (393)
Jiading district	17.2% (394)
Chongming district	18.8% (431)
Yangpu district	12.6% (289)
Xuhui district	16.9% (387)
Pudong district	17.3% (397)

Lead levels by gender were shown in Table 2. Boys had significantly higher lead levels than those of girls. The positive rate of lead concentrations ≥100 µg/L for boys and girls were 0.34% (4/1186) and 0.18% (2/1105), there is no significant difference between sex (χ2 = 1.214, p>0.05). The positive rate of lead concentrations ≥50 µg/L for boys and girls were 2.8% (33/1186) and 2.5% (28/1105), there is no significant difference between sex (χ2 = 1.208, p>0.05).

**Table 2:** Difference in lead level by gender (µg/L).

Sex	Number	Arithmetic Mean	Geometric Mean	Median	Range
Boy	1186	23.57	20.60^a^	21	3–246
Girl	1105	21.2	18.41	18	3–89
χ2	6.285				
P	0.002				

a boys are higher than girls, p<0.001;

Lead levels by ages were shown in Table 3. The blood lead levels were significantly increased with the age, and the 3 to 6 year-old was the highest in all ages.

**Table 3:** Difference in lead level by ages (µg/L).

Ages	Number	Arithmetic Mean	Geometric Mean	Median	Range
0–11 m	221	19.32	16.38	17	3–59
12–35 m	710	21.17	18.38^a^	19	3–122
36–72 m	1360	23.84	20.95^a^ ^b^	21	4–246
χ2	35.06				
P	0.000				

a the age of 1 to 3-year-old is higher than 0 to 1-year-old, p<0.001;

b the age of 3 to 6-year-old is higher than 1 to 3-year-old, p<0.001.

**Figure 1:**
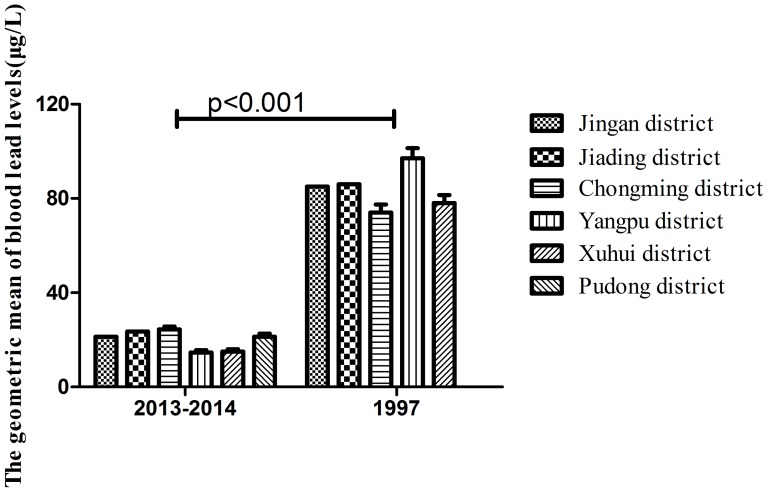
**Figure 1** showed that the blood lead level of this survey was significantly lower than the blood lead level of 1997. This survey showed that the Chongming district was the highest and Yangpu district was the lowest. 1997's survey showed that Yangpu district was the highest among all the five districts, and the Chongming district was the lowest.

The geometric mean of blood lead levels of Jingan, Jiading, Chongming, Yangpu and Xuhui districts were significantly lower than the data in 1997 of Shanghai. 1997's survey showed that Yangpu district was the highest among all the five districts, and the Chongming district was the lowest. This survey showed that the Chongming district was the highest and Yangpu district was the lowest. (see in Table 4, Fig. 1)

**Table 4:** Difference in lead level by districts (µg/L).

Districts	Number	Geometric	Number	Geometric	p
	2013–2014	Mean	1997	Mean	
Jingan district	289	21.32	441	85^a^	0.000
Jiading district	431	23.54	369	86^a^	0.000
Chongming district	394	24.41	413	74^a^	0.000
Yangpu district	397	14.67	359	97^a^	0.000
Xuhui district	387	15.03	387	78^a^	0.000
Pudong district	393	21.37	0	0	0.000

a the blood lead level of 1997 is higher than now, p<0.001;

Eating Chinese traditional popcorn, smoking by the main caregivers, presence of a small family workshop nearby, caregivers' degree of education, eating fried food and the family engaged in a lead related occupation were risk factors for low blood levels of lead (Logistic regression analysis OR 47.87, 3.43, 4.95, 1.25, 1.24, and 1.25, respectively). Zinc supplementation and washing hands with soap before eating were protective factors (OR, 0.36 and 0.33, respectively). (see in Table 5)

**Table 5:** Factors affecting blood lead levels (Logistic regression analysis).

Factors	β	Wald χ2	P	OR(95%Cl)
Eating popcorn	3.89	16.08	0.000	47.87(7.23,317.01)
Zinc supplementation	−1.01	4.28	0.039	0.36(0.14,0.95)
Smoking by main caregivers	1.23	4.75	0.029	3.43(1.13,10.40)
Small family workshop nearby	1.60	8.45	0.004	4.95(1.68,14.55)
Caregivers' degree of education	2.28	21.23	0.000	1.25(1.14,1.37)
Washing hands with soap before eating	0.89	3.79	0.032	0.33(0.09,0.89)
Eating fried food	0.79	5.32	0.001	1.24(1.08,1.54)
Family engaged in a lead related occupation	1.56	6.25	0.017	1.25(1.202,3.63)

## Discussion

Childhood lead poisoning is a worldwide preventable problem. The average lead blood level of 728 children in Belgium from 1991 to 1995 was reported as 87 µg/L. The mean lead blood level of 269 children in Singapore from 1995 to 1997 was reported as 66 µg/L [11]. In 1991 American [12] evaluated 2234 children aged 1 to 5 years old and found a mean blood lead level of 36 µg/L. The mean lead blood level of 1109 children in Canada from 1993 to 1995 was reported as 15.7 µg/L [13]. We found the arithmetic mean, geometric mean and median of blood lead levels of 0 to 1-year-old,1 to 3-year-old and 3 to 6-year-old children from Shanghai were 22.49 µg/L, 19.65 µg/L and 19.5 µg/L, including 0.2% (4/2291) with concentrations ≥100 µg/L and 2.4% (46/2291) with concentrations ≥50 µg/L. This value was lower than that reported by the Centers for Disease Control and Prevention in China in 2001, a total of 6502 children from 9 provinces and 19 cities were evaluated and found to have a mean lead blood level of 88.3 µg/L. The prevalence of lead poisoning was reported as 29.91% [14]. Despite the decline in children's lead levels, lead poisoning is still main public health problem.

We found that boys' mean lead levels (23.57 µg/L) were higher than that of girls (21.2 µg/L) so as the survey did by the third national United States Health and Nutrition. The results of our study are consistent with it. These differences may be related to greater autonomous behavior and outdoor activities found with boys as they grew, leading to contact with environmental lead pollution. Boys may have a greater need for supervision, health education, and health care.

This survey showed the blood lead levels were significantly increased with the age, and the 3 to 6 year-old was the highest in all ages. Many studies show that the exposure risks are staggered and mixed with aging, BLLs change with the passage of time, usually in 1 to 3 years old appears BLLs peak, because of the child may walk independently, as well as hand to mouth moves increased. S Wang's [15] survey also demonstrate the same result with our survey.

This survey showed that the geometric mean of Jingan, Jiading, Chongming, Yangpu and Xuhui districts were significantly lower than the data in 1997 of Shanghai. 1997's [9], [10] survey showed that Yangpu district was the highest among all the five districts, and the Chongming district was the lowest. This survey showed that the Chongming district was the highest and Yangpu district was the lowest. Yangpu district was an old industrial area in 1997, there were factories such as lead smelters and other lead related industries, so the highest blood lead levels of children was found in Yangpu district, Chongming is a relatively suburban district, and there was no industrial development, so it was at a low blood lead at that period of time. With the introduction of lead free gasoline and industrial transformation in Shanghai since 1997, Shanghai's industrial structure has also changed, many industrial factories moved to the outskirts of the city, as well as pollutant sources have moved to the suburbs outside. So as this survey showed, Yangpu district as a central district nowadays, the blood lead level was the lowest, and Chongming district is a new industry district, its blood lead level was the highest of all.

Chinese traditional popcorn is quite popular among folks. Chinese traditional popcorn is rich in lead because of its particular productive processing. Due to the low melting point of lead, the sealing layer of Chinese traditional popcorn machine contains lead, which would melt at a relatively low temperature to guarantee the tightness of machine during popcorn productive processing. High temperature releases lead vapors, finally increased the lead level in Chinese traditional popcorn.

Factories close to residential areas can produce industrial pollutants and lead dust spills into nearby residential areas, contaminating the local residents. A variety of such industries, including battery manufacturing, metal smelting, printing, mechanical manufacturing, and ship building, are present in China. Lead dust is inhaled and ingested via children's unwashed hands. Hand washing can eliminate the digestive component of lead dust contamination [16]. Another important source of lead exposure for children is from toys and lead paints. Other oral sources of lead include ingestion of preserved eggs, and fried food.

Parents' level of education can indirectly affect children's blood lead level. Parents' learning and understanding of lead poisoning is related to their level of education. We found that parents with less than high school degree more frequently had children with higher blood lead levels. In addition, parents engaged in a lead related occupation were also at risk of having children with increased blood lead levels, presumably due to lead dust carried from the workplace to home. Otherwise, cigarette smoke contains lead dust, parents' smoking brings lead to children as well. Thus, Greater awareness of environmental risk factors is needed.

Iron, zinc, calcium, and lead are all divalent metal ions, and are absorbed from the gastrointestinal tract and metabolized through common pathways. Dietary deficiencies of the trace elements iron, zinc, and calcium will lead to increased lead absorption. Similarly, increased absorption of lead can also lead to a reduction in the absorption of iron, zinc, and calcium. A diet full of dairy products can act to diminish the absorption of lead.

Blood lead screening is the only effective way to identify lead-poisoned children. Every year, tens of thousands of children are screened in China, and a considerable number of children with elevated blood lead levels and lead poisoning are identified. However, screening for blood lead occurs typically in response to requests from parents and not as part of common overall examination or policy. Thus, a large number of children may have lead poisoning that are undetected, and do not receive timely diagnosis or treatment. Routine physical examination cannot detect childhood lead contamination.

A number of policies and measures should be implemented to promote the prevention and control of childhood lead poisoning in China. Regulatory policies need to be put in place to reduce lead emissions from the numerous lead-related industries. There is a need to improve blood lead screening and blood lead testing, and to initiate a nationwide implementation of standardized blood lead testing techniques and methods. Every child ≤6 years of age should have the opportunity to receive blood lead testing. Special screening programs should also be developed for children living in lead-contaminated areas. Finally, the public and pediatricians in China need to be educated about the prevention and treatment of childhood lead poisoning.

This was the first study to compare the lead levels among different years and to examine the relationship between exposure risk factors and blood lead levels in children of Shanghai. The sampling method design was one of the primary advantages of this study. However, there were some limitations to this study. First, the lead concentrations were determined at only one point in time, making it difficult to know about the degree to which our results reflect the circumstances of lead exposure throughout the whole period of childhood development. Second, we did not evaluate the levels of other hazardous environmental pollutants (e.g., Hg, cadmium, and so on) or of prenatal nutrients, which may act as confounders to our analysis. Third, ours was a relatively small study. A larger study is needed to verify our results.
